# Ethics and Epidemics

**DOI:** 10.3201/eid1009.040407

**Published:** 2004-09

**Authors:** Robert Baker, Wayne Shelton, Martin Strosberg

**Affiliations:** * Graduate College of Union University, Schenectady, New York, USA;; † Albany Medical College, Albany, New York, USA

**Keywords:** bioethics, biomedical ethics, healthcare ethics, ethicists, medical, human rights, disease outbreaks, communicable diseases, quarantine, communication

More than 90 people attended a March 25–27, 2004, conference on Ethics and Epidemics. This conference was sponsored by the Albany Medical College–Graduate College of Union University Masters in Bioethics Program, the University at Albany School of Public Health, the New York State Department of Health, and the Wadsworth Laboratories. Attendees came from Australia, Africa, Asia, Europe, Canada, and the United States. Among the 24 papers and panels, presentations were made by George Annas, professor and chair of Health Law at the Boston University School of Public Health; Ezekiel Emanuel, chair of the Department of Clinical Bioethics of the Magnuson Center of the National Institutes of Health; Thomas R. Freiden, commissioner, New York City Department of Health; Matthew Wynia, director of the Institute of Ethics of the American Medical Association; Kenyan bioethicist Angela Wassuna associate for International Affairs of the Hastings Center; and 19 other bioethicists and health professionals.

Presentations ranged from case studies to health policy debates. Many reviewed the history of epidemics, emphasizing their global nature and the imperative of global strategies for epidemic control. Several papers examined recent epidemics and explored new strategies for dealing with epidemic control while respecting human rights. The consensus was that the old policeman model of public health needs updating. Discussion focused on how best to balance public safety, professional responsibility, personal liberty, and human rights, while effectively containing epidemics. Emanuel and Wynia reaffirmed the responsibility of physicians and first responders to put their health and lives at risk in combating epidemics. Yet, noting the vulnerability of first responders (in the Toronto severe acute respiratory syndrome [SARS] outbreak and elsewhere), they distinguished between bravery and foolhardiness, arguing that just as professionals have a responsibility to protect the public from disease, the public, in turn, has a responsibility to provide the training, equipment, and resources to minimize the need to take risks.

Virtually all conferees observed that the public health infrastructure needs substantial rebuilding to cope effectively with epidemics. Annas, however, noted that in bioterrorist assaults the control of biologic agents is a public health problem to be dealt with by public health officials, not by the U.S. Department of Defense or the U.S. Department of Homeland Security. He further stated that policies on epidemic control that involve consistent, open, and truthful communication with the public—like those used in New York and Toronto during the recent SARS outbreak—create cooperative environments that minimize conflicts between freedom and safety and limit the effects of isolation and quarantine. However, Emanuel et al. asserted that the traditional enforcement authority of public health law was essential and needed as a fallback. The result of the debate was that 21st century methods need to be developed to control infectious disease epidemics that reconcile the need to protect public health and respect human rights.

The conference program is available on http://www.bioethics.union.edu under "News." For further information contact bioethics@union.edu or 518-388-8045.

